# Tunneling nanotubes evoke pericyte/endothelial communication during normal and tumoral angiogenesis

**DOI:** 10.1186/s12987-018-0114-5

**Published:** 2018-10-05

**Authors:** Mariella Errede, Domenica Mangieri, Giovanna Longo, Francesco Girolamo, Ignazio de Trizio, Antonella Vimercati, Gabriella Serio, Karl Frei, Roberto Perris, Daniela Virgintino

**Affiliations:** 1 Department of Basic Medical Sciences, Neurosciences, and Sensory Organs, Human Anatomy and Histology Unit, University of Bari School of Medicine, Bari, Italy; 2 Department of Medical and Surgical Sciences, Biomedical Unit ‘E. Altomare’, University of Foggia, Foggia, Italy; 3 Department of Basic Medical Sciences, Neurosciences, and Sensory Organs, Molecular Biology Laboratory, University of Bari School of Medicine, Bari, Italy; 4 Department of Neurosurgery, Neurocenter of Southern Switzerland, Regional Hospital Lugano, Lugano, Switzerland; 5 Department of Biomedical Sciences and Human Oncology, University of Bari School of Medicine, Bari, Italy; 6 Department of Emergency and Organ Transplantation, Division of Pathology, University of Bari School of Medicine, Bari, Italy; 7 Department of Neurosurgery, University Hospital Zurich, Zurich, Switzerland; 8 COMT-Centre for Molecular and Translational Oncology & Department of Chemical and Life Sciences and Environmental Sustainability, University of Parma, Parma, Italy

**Keywords:** Tunneling nanotubes, Pericytes, Cell-to-cell communication, Angiogenesis, Developing cerebral cortex, Glioblastoma

## Abstract

**Background:**

Nanotubular structures, denoted tunneling nanotubes (TNTs) have been described in recent times as involved in cell-to-cell communication between distant cells. Nevertheless, TNT-like, long filopodial processes had already been described in the last century as connecting facing, growing microvessels during the process of cerebral cortex vascularization and collateralization. Here we have investigated the possible presence and the cellular origin of TNTs during normal brain vascularization and also in highly vascularized brain tumors.

**Methods:**

We searched for TNTs by high-resolution immunofluorescence confocal microscopy, applied to the analysis of 20-µm, thick sections from lightly fixed, unembedded samples of both developing cerebral cortex and human glioblastoma (GB), immunolabeled for endothelial, pericyte, and astrocyte markers, and vessel basal lamina molecules.

**Results:**

The results revealed the existence of pericyte-derived TNTs, labeled by proteoglycan NG2/CSPG4 and CD146. In agreement with the described heterogeneity of these nanostructures, ultra-long (> 300 µm) and very thin (< 0.8 µm) TNTs were observed to bridge the gap between the wall of distant vessels, or were detected as short (< 300 µm) bridging cables connecting a vessel sprout with its facing vessel or two apposed vessel sprouts. The pericyte origin of TNTs ex vivo in fetal cortex and GB was confirmed by in vitro analysis of brain pericytes, which were able to form and remained connected by typical TNT structures.

**Conclusions:**

None of the multiple roles described for TNTs can be excluded from a possible involvement during the processes of both normal and pathological vessel growth. A possible function, suggested by the pioneering studies made during cerebral cortex vascularization, is in cell searching and cell-to-cell recognition during the processes of vessel collateralization and vascular network formation. According to our results, it is definitely the pericyte-derived TNTs that seem to actively explore the surrounding microenvironment, searching for (site-to-site recognition), and connecting with (pericyte-to-pericyte and/or pericyte-to-endothelial cell communication), the targeted vessels. This idea implies that TNTs may have a primary role in the very early phases of both physiological and tumor angiogenesis in the brain.

**Electronic supplementary material:**

The online version of this article (10.1186/s12987-018-0114-5) contains supplementary material, which is available to authorized users.

## Background

In 2004, Amin Rustom, Hans-Hermann Gerdes and Colleagues described in the journal Science [1], “highly sensitive nanotubular structures formed de novo between pheochromocytoma cultured cells, that create complex networks” as a sort of “highways” dedicated to cell-to-cell communication. Actually, it was at the very beginning of the century that Amin Rustom observed these long, delicate cell strands for the first time, owing to a mistake in culture protocol, and named them tunneling nanotubes (TNTs) (see Anil Ananthaswamy, https://www.sott.net/article/170469-Tunnelling-nanotubes-Lifes-secret-network and Viviane Callier, https://www.quantamagazine.org/cells-talk-and-help-one-another-via-tiny-tube-networks-20180423/). Although TNTs have been described in recent times, long pseudopodial processes were firstly described by Thomas Bär [2] as structures connecting facing, growing microvessels, “…filopodia may be responsible for the establishment of intervascular bridges which later become canalized” (part of Bär’s original diagram is shown in Additional file 1). Additional structural and functional characteristics of TNTs have subsequently been revealed by a number of studies (published from 2006 to 2008) carried out on different cell types in vitro [3–6]. TNTs were demonstrated to accomplish long-range, directed communication between dislodged cells and to mediate intercellular transfer of diverse molecules and cell components [3]. They were described as having an average length of 30 µm and in some case above 140 µm, and have been grouped into two main classes, very thin (≤ 0.7 µm, measuring a minimum of 100–200 nm) and thick (≥ 0.7 µm, up to 1 µm) [4]. TNTs heterogeneity does not only depend on their extension and diameter but is also due to differences in their own molecular features [5, 6] like, for instance, the inconstant presence of actin microfilaments, gap junctions and lipid raft-type membrane microdomains [1, 7–9]. Two main classes of TNTs have been described, according to their different morphology/function: TNTs type I, short dynamic structures, containing actin filament and engaged in exploring the surrounding microenvironment, and TNTs type II, that are longer and more stable processes, containing cytokeratin filaments and apparently involved in organelles shuttle [6].

The history of TNTs discovery testifies to their extreme fragility; in fact, they are very sensitive structures that can be damaged by light exposure, shearing force and chemical fixation [1], consistent traits that have hampered TNTs histological observation in ex vivo tissues. Hence the prevalence of in vitro studies that have investigated TNTs biological significance and molecular composition. The first evidence of the existence of TNTs in tissues, in vivo, was elegantly demonstrated and documented by Chinnery, Pearlman, and McMenamin in 2008, when they described TNTs in the corneal stroma of chimeric mouse, between donor-derived MHC class II^+^ cells and resident MHC class II^+^ cells [10]. More recently, TNT-like structures in vivo have been described in human pleural mesothelioma and adenocarcinoma [11], in cultured explants of ovarian cancer [12], and in solid samples of laryngeal squamous cell carcinoma [13]. Our knowledge of TNT function has been improved by studies demonstrating the involvement of TNTs in Herpes viruses intercellular spread [14, 15], in myeloid cell communication in immune surveillance [16], and their use as ‘emergency highways’ for the transport of vital organelles during situations posing a risk of apoptosis in damaged cells [17]. In addition, TNT communication via chemical messengers and/or the transfer of organelles between malignant cells involved in cancer growth, invasion and metastasis has recently gained in importance, revealing TNTs as a promising target in cancer therapy [18, 19]. Obviously this concept can be extended to other diseases, such as neurodegenerative diseases, in which a TNT pathogenesis has been suggested [20, 21]. To date, only few publications describing the existence of TNTs in the central nervous system have been published and, to the best of our knowledge, no studies have investigated ‘vascular’ TNTs during physiological and tumor angiogenesis in the brain.

**Fig. 1:**
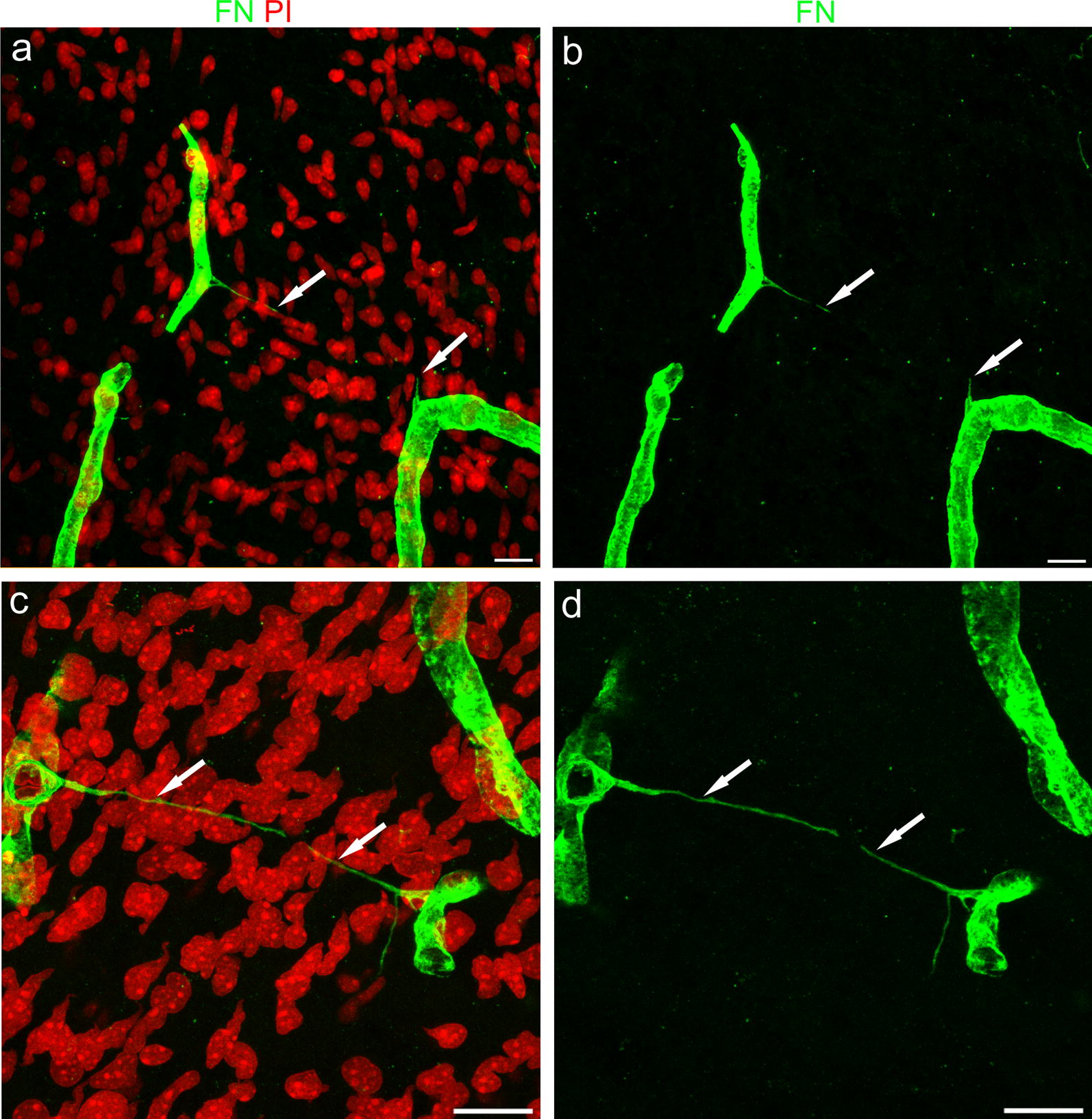
Representative confocal microscopy images of TNTs in the developing human telencephalon at 18 wg. **a**, **c** Fibronectin (FN) staining reveals the basal lamina of cerebral cortex microvessels and FN^+^ TNTs (arrows). **b**, **d** On the single FN channel (green) the FN^+^ TNT in (**b**) appears interrupted (arrows), while in **d** a long and fine FN^+^ TNT (arrows) bridges the gap between two distant vessels. Nuclear counterstaining propidium iodide (PI). Scale bars **a**–**d** 25 µm

We firstly observed TNTs in the developing human telencephalon at 18 wg, during Bär’s second stage of intracortical vascularization, a phase featuring cerebral cortex vascular network formation and characterized by a rapid increase in vessel branching and vessel density [2, 22, 23]. At this time, TNTs were identified on slides immunostained with fibronectin (FN), one of the major molecular components of the vessel basal lamina (VBL). On these fields, TNTs were revealed as tiny structures, stretched between two microvessels, but were often interrupted and difficult to follow over their entire length (Fig. 1a, b). A more appropriate tissue processing, with lighter fixation and amplified immunostaining systems applied to thick, free-floating sections, as well as overexposed laser confocal microscope settings, improved the TNTs structural preservation and detection, allowing us to observe very long, tiny TNTs bridging facing microvessels (Fig. 1c, d). Additional examples of straight and spiraled, non-bridged FN^+^ TNTs are shown in Additional files (Additional file 2a–f).

These observations opened out the vista of a possible involvement of TNTs in vessel growth and cell-to-cell communication during brain vascularization. Following up on this idea, we investigated the cellular ‘source(s)’ of TNTs in the microvessel wall by laser confocal immunofluorescence, using antibodies against specific cellular (endothelial cells, pericytes, and perivascular astroglia) and molecular (collagen type IV and VI) microvessel components. The analysis was carried out on cerebral cortex samples from human fetuses at midgestation (18 and 22 weeks of gestation, wg) and on human glioblastoma (GB) specimens, as an example of pathological angiogenesis in highly vascularized brain tumors. Based on these results in tissue ex vivo, culture assays were added to explore the formation of TNTs in vitro.

Tissue and culture results indicate pericytes as the main source of TNTs during the processes of vessel growth and branching in both developing brain and GB, suggesting that these cells are involved in neuroangiogenesis and in tumoral angiogenesis preventive recognition and communication steps, through their active, vascular TNT wiring.

## Methods

### Fetal specimen histology and immunostaining

Autopsy specimens of fetal brain were collected from fetuses at 18 and 22 weeks of gestation (wg; 2 for each of the examined ages) spontaneously aborted due to preterm rupture of the placental membranes and with no history of neurological pathologies. Permission to collect fetal tissue was obtained from the mother at the end of the abortion procedure. The sampling and handling of the specimens conformed to the ethical rules of the Department of Emergency and Organ Transplantation, Division of Pathology, University of Bari School of Medicine, and approval was gained from the local Ethics Committee of the National Health System in compliance with the principles stated in the Declaration of Helsinki. The fetal age was estimated based on the crown-rump length and/or pregnancy records (counting from the last menstrual period). At autopsy, the fetuses did not reveal macroscopic structural abnormalities and/or malformations of the central nervous system. The dorso-lateral wall of each telencephalic vesicle (future cerebral cortex), was dissected, along the coronal planes, in samples (n = 6) about 0.5 cm thick, fixed for 2–3 h at 4 °C by immersion in 2% paraformaldehyde (PFA) plus 0.2% glutaraldehyde in phosphate-buffered saline solution (PBS, pH 7.6) and then washed in PBS. For each fetus, 3–4 samples were serially cut using a vibrating microtome (Leica Microsystem; Milton Keynes, UK) into 20-µm sections, parts of which were processed for conventional histological analysis with toluidine blue staining to ascertain the absence of microscopic malformations. All the other sections were stored at 4 °C in PBS plus 0.02% PFA for immunolabeling and fluorescence microscopy. Single and multiple immunostainings were carried out with the following polyclonal (pAb) and monoclonal (mAb) antibodies diluted in blocking buffer (BB; 1% bovine serum albumin and 2% fetal calf serum in PBS): pAb anti-fibronectin (Dako, Glostrup, Denmark), mAb anti-CD31 (Dako), pAb anti-CD105 (Abcam, Cambridge, UK), pAb anti-Glut-1 (Santa Cruz Biotechnology, Santa Cruz, CA, USA), mAb anti-CD146 (Abcam), pAb anti NG2/CSPG4 D2 (gift from William B. Stallcup, The Burnham Institute for Medical Research, La Jolla, CA, USA), mAb anti-collagen type IV (Dako), pAb anti-collagen type IV (Acris Antibodies GmbH; Herford, Germany), pAb anti-collagen type VI (Abcam), and mAb anti-GFAP (Vision Biosystem Novocastra, Newcastle upon Tyne, UK) (Table 1). Free-floating sections were incubated with 0.5% Triton X-100 in PBS for 30 min at room temperature (RT), BB 30 min at RT, followed by incubation, with single or combined primary antibodies, overnight at 4 °C and with appropriate fluorophore-conjugated secondary antibodies (fluorophore 488, 555, and 633; Thermo Fisher Scientific; Waltham, MA, USA) or biotinylated secondary antibodies for 45 min at RT, the latter subsequently revealed by fluorophore-conjugated streptavidin (Streptavidin-Alexa 488, Streptavidin-Alexa 555; Thermo Fisher Scientific) (Table 1). After each incubation step the sections were washed 3 times for 5 min with PBS. The sections were then post-fixed in 4% PFA for 10 min and nuclear counterstaining was performed by incubations either in RNase (diluted 5 µl/ml in PBS; Thermo Fisher Scientific) and then propidium iodide (PI; diluted 1 µl/ml in PBS; Thermo Fisher Scientific) or by TOPRO-3 or SYTOX (diluted 1:10 K in PBS; Thermo Fisher Scientific). Finally, the sections were allowed to adhere on polylysine slides (Menzel-Glaser, GmbH, Braunschweig, Germany) by drying for 10 min at RT, were coverslipped with Vectashield (Vector Laboratories Inc., Burlingame, CA, USA), and sealed with nail varnish. Negative controls were prepared by omitting the primary antibodies and by mismatching the secondary antibodies.

**Table 1:** List of primary and secondary antibodies

Primary antibodies	Host IgG	Dilution	Concentration	Producer	Cat. no.
Fibronectin	Rabbit IgG	1:100	5 mg/ml	Dako	A0245
CD31	Mouse IgG_1k_	1:10	0.205 mg/ml	Dako	M0823
CD105	Rabbit IgG	Prediluted	NA	Abcam	AB27422
Glut-1	Goat IgG	1:100	0.2 mg/ml	Santa Cruz	sc-1605
CD146	Mouse IgG_1_	1:50	0.123 mg/ml	Abcam	AB49492
NG2/CSPG4 D2	Rabbit IgG	1:50	NA	W.B. Stallcup	–
Col type IV	Mouse IgG_1k_	1:100	0.5 mg/ml	Dako	M0785
Col type IV	Rabbit IgG	1:100	1 mg/ml	Acris	R1041
Col type VI	Rabbit IgG	1:100	1 m/ml	Abcam	AB6588
GFAP	Mouse IgG_1_	1:100	0.1 mg/ml	Monosan	MONX10749
NG2/CSPG4	Mouse IgG	1:200	0.5 mg/ml	Thermo Fisher	14-6504-80

Secondary antibodies		Dilution	Concentration	Producer	Cat. no.
Biotinylated goat-anti rabbit		1:400	1.5 mg/ml	Vector	BA-1000
Streptavidin-Alexa 488		1:300	2 mg/ml	Thermo Fisher	S-11223
Streptavidin-Alexa 555		1:300	2 mg/ml	Thermo Fisher	S-21381
Goat anti mouse IgG Alexa 488		1:300	2 mg/ml	Thermo Fisher	A11001
Goat anti mouse IgG_1_ Alexa 555		1:300	2 mg/ml	Thermo Fisher	A21127
Goat anti mouse IgG_1_ Alexa 633		1:300	2 mg/ml	Thermo Fisher	A21126
Goat anti-rabbit Alexa 488		1:300	2 mg/ml	Thermo Fisher	A11070
Donkey anti-goat Alexa 488		1:300	2 mg/ml	Thermo Fisher	A11055
Goat anti-rabbit Alexa 555		1:300	2 mg/ml	Thermo Fisher	A32732

*NA* not available

### Glioblastoma histology and immunostaining

Glioblastoma (GB) samples (n = 6) were collected from primary tumors specimens obtained in a previous study, in which the imaging techniques and histological analysis were specified in detail [24]. The six GB patients were males (n = 4) aged from 30 to 55 years-old, and females (n = 2), of 52 and 53 years-old, who underwent surgery at the Department of Neurosurgery, University Hospital Zurich (Switzerland). Written informed consent was obtained from patients before study entry. All procedures were approved by the Ethics Committee of the University of Bari Medical School and by the Ethics Committee of Canton Zurich, in accordance with the Declaration of Helsinki. Glioma samples were classified according to the WHO 2007 criteria. The samples were dissected (≤ 0.5 cm in thickness) and then fixed for 2–3 h at 4 °C by immersion in 2% PFA plus 0.2% glutaraldehyde in phosphate-buffered saline solution (PBS, pH 7.6). Specimens were then washed in PBS, and serially cut using a vibrating microtome (Leica Microsystem; Milton Keynes, UK); 20-μm sections were stored at 4 °C in PBS plus 0.02% PFA for immunolabeling and fluorescence microscopy. Double immunostainings were carried out with mAb anti-CD31 and pAb anti-collagen type IV, as described for fetal sections. Negative controls were prepared by omitting the primary antibodies and by mismatching the secondary antibodies.

### Laser confocal microscopy analysis and measurements

Sections were examined with a Leica TCS SP5 confocal laser-scanning microscope (Leica Microsystems, Mannheim, Germany) using a sequential scanning procedure and, when appropriate, an overexposed laser setting. Confocal images were taken at 0.35 µm intervals through the z-axis of the sections, with 40× and 63× oil lenses associated to zoom factors from 1.5 to 3. Single, serial optical planes and z-stacks (projection images) were analyzed by Leica confocal software (Multicolour Package; Leica Microsystems). The size of TNT-like structures was evaluated with LAS-AF SP5 software (Leica Microsystems) on 63× magnification fields zoomed 3 times. TNT thickness (µm) was measured on projection images from fetal cerebral cortex (n = 4), stained for NG2, for a total of 63 TNT fields. The results are expressed as mean ± standard deviation (M ± SD) together with the maximum (Max) and minimum (Min) values.

### Pericyte tunneling nanotube assays

Human brain vascular pericytes (HBVP) were purchased from CellScience (CellScience, Research Laboratory, Carlsbad, CA, USA) and cultured in Pericyte Culture Medium (PCM), supplemented with 2% fetal bovine serum; Pericytes Growth supplement; 2 mM l-Glutamine and antibiotics (100 U of penicillin G and 100 μg/ml of streptomycin sulphate). Cell cultures were maintained at 37 °C in a humidified 5% CO_2_ atmosphere. At confluence, HBVPs were detached with Accutase (GE Healthcare) and resuspended in complete PCM, then 5 × 10^4^ HBVP were seeded on Matrigel layer and cells were incubated at 37 °C for 5 h. Then medium from each well was gently aspirated and cells were fixed with 100 µl of 4% PFA at 4 °C overnight. The PFA solution was then gently removed and the cells were maintained in PBS containing 0.02% PFA. The relevant in vitro observations were carry out with HBVP at passage 3. The formation of TNTs was documented with a microscope (Eclipse TS100, Nikon Italia) equipped with a CCD camera (DS-Qi1Mc; Nikon Italia), and their diameter was estimated using Nikon NIS software on 20× magnification fields zoomed 3 times. A total of 25 fields was evaluated to assess the average thickness of TNTs. For immunofluorescent staining, HBVP were seeded on glass coverslips pre-coated with gelatine and allow to adhere for 24 h, then fixed in 4% PFA at RT for 20 min and permeabilized with 0.5% Triton X-100 in PBS for 5 min. The cells were incubated with the following reagents: Phalloidin TRITC-conjugated (1:500 in PBS, ECM-Biosciences, Versailles, USA; code PF7551), or Lipophilic Cell Tracker Dil (1:200 in PBS, Invitrogen, code C7001). The cells were immunostained with mAb anti-Neural/Glial Antigen2/Chondroitin sulfate proteoglycan 4 (NG2/CSPG4, Thermo Fisher Scientific) overnight at 4 °C, revealed by an anti-mouse fluorophore 488-conjugated secondary antibody (Thermo Fisher Scientific). After each incubation step the sections were washed 3 times for 5 min with PBS. The glasses were mounted on Vectashield containing DAPI (Invitrogen) diluted 1 mg/ml in distilled water and images were taken at 20× magnification with an inverted epifluorescence microscope (Zeiss Axio Observer Z1; Carl Zeiss Microscopy, Oberkochen, Germany) equipped with a CCD camera (LMS710, Zeiss).

## Results

### TNTs in human developing cerebral cortex

**Fig. 2:**
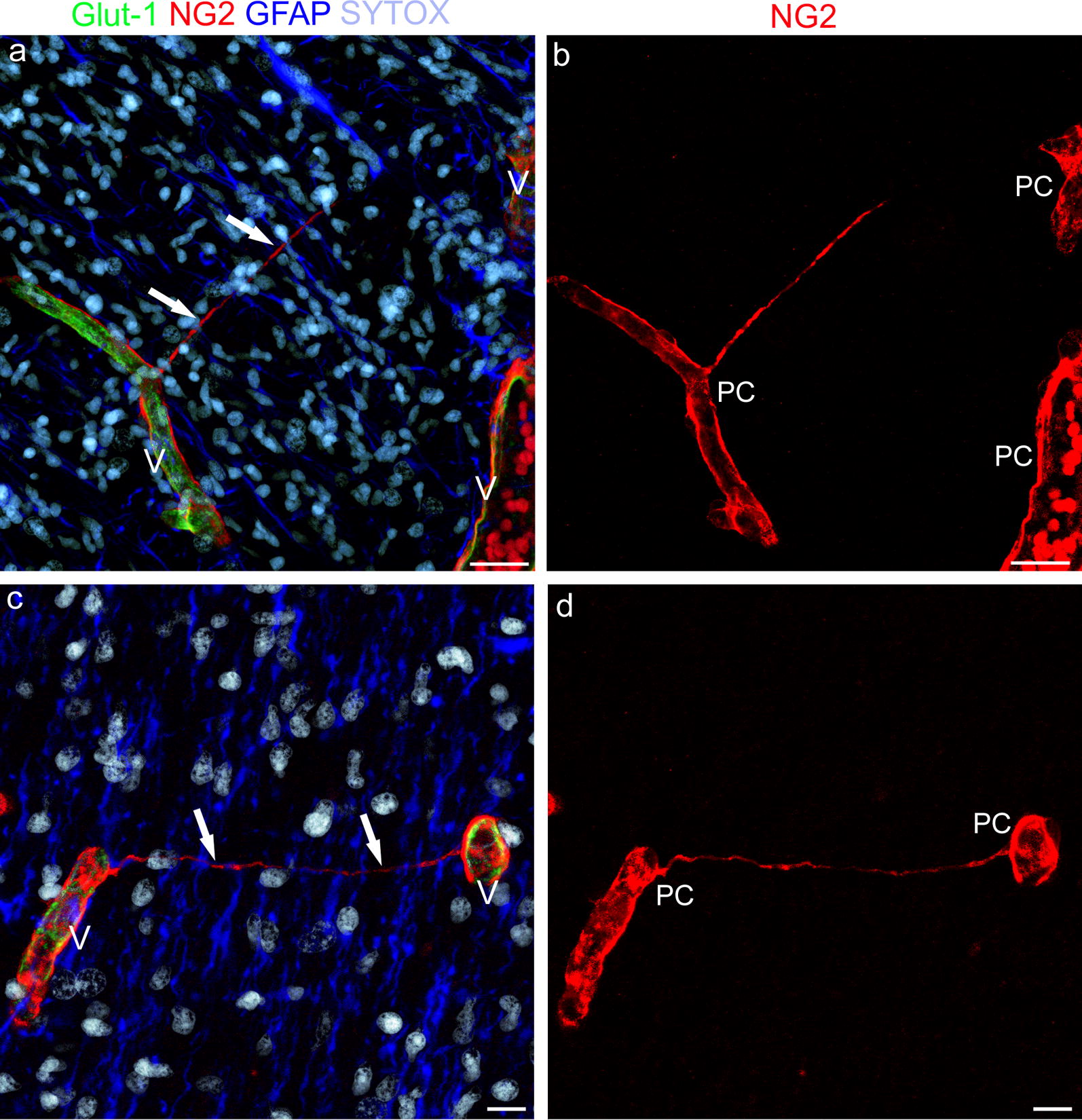
Representative confocal microscopy images of blood–brain barrier (BBB)-microvessels and TNTs in the developing human telencephalon at 22 wg. **a**, **c** NG2^+^, pericyte TNTs (arrows) originate from cortex microvessels (V) lined by Glut-1^+^ BBB-endothelial cells, and pass through a curtain of GFAP^+^ radial glia fibers; **b**, **d** The single channel (red) better shows NG2^+^ pericytes (PC), which cover the microvessel wall, together with the finer details of their NG2^+^ TNTs. Nuclear counterstaining SYTOX. Scale bars **a**, **b** 25 µm; **c**, **d** 20 µm

**Fig. 3:**
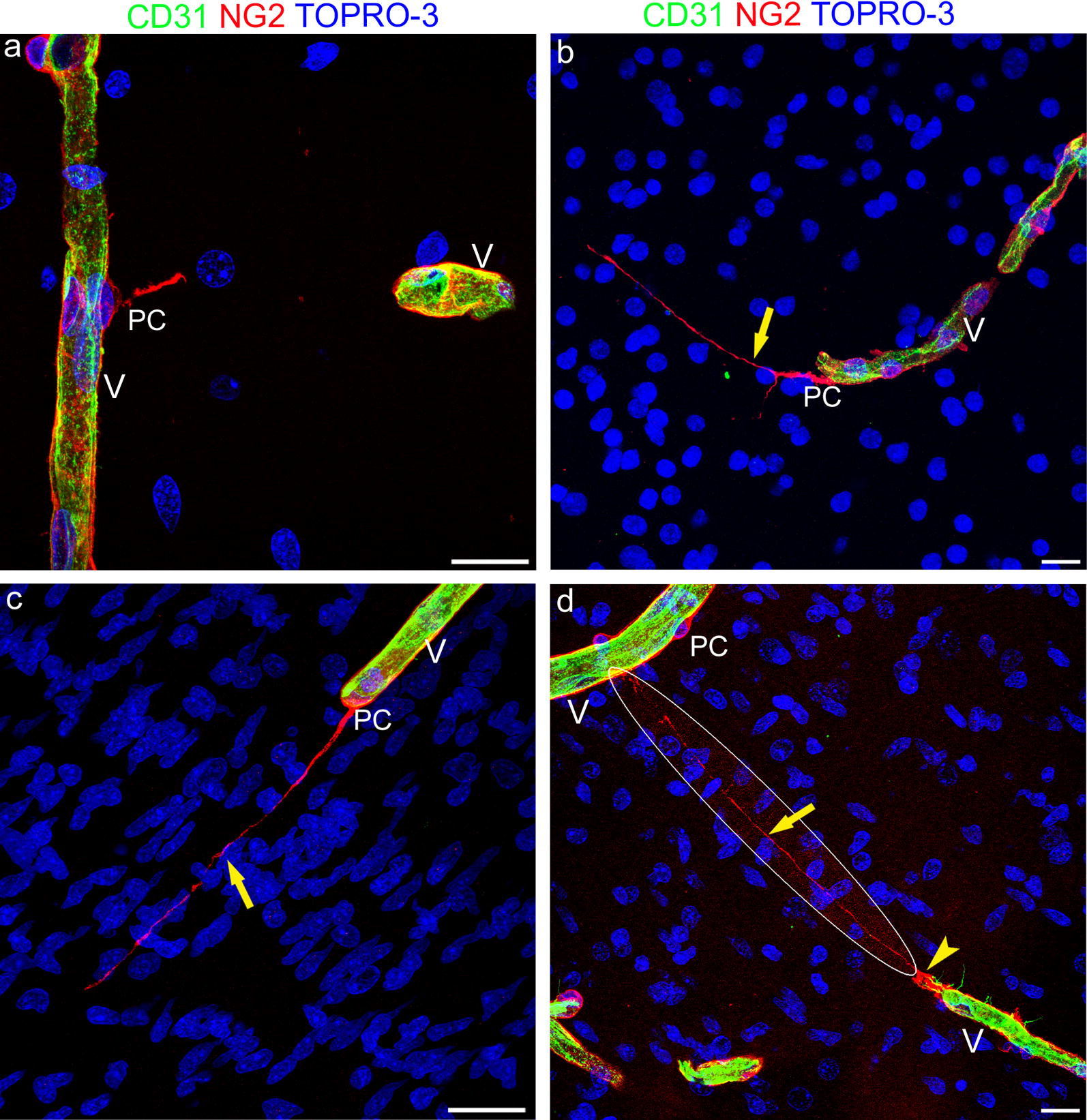
Representative confocal microscopy images of pericyte TNTs in the developing human telencephalon at 22 wg. **a** Two microvessels (V) revealed by CD31^+^ endothelial cells and NG2^+^ activated pericytes (PC). **b**, **c** Bent (**b**, arrow) and straight (**c**, arrow) NG2^+^ TNT originate from pericytes (PC) associated with microvessels (V) lined by CD31^+^ endothelial cells. **d** A nearly undetectable, very thin and ultra-long NG2 TNT (arrow) connects a growing microvessel (V), escorted by a leading pericyte (arrowhead), to the recipient vessel (V); the encircled area corresponds to an overexposed laser confocal microscope setting. PC pericyte. Nuclear counterstaining TOPRO-3. Scale bars **a**–**d** 20 µm

Triple immunostainings with Glut-1, as a marker of blood brain barrier (BBB)-endothelial cells (ECs), the pericyte marker NG2 proteoglycan, and GFAP for astroglia cells, were carried out to analyze the possible cellular source(s) of TNTs in the developing cerebral cortex (Fig. 2a–d). On these sections, apposed microvessels lined by Glut-1^+^ BBB-ECs, and typically covered by NG2^+^ pericytes, appeared embedded in a parenchyma characterized by curtains of GFAP^+^ astroglia cells (mainly radial glia fibers) and densely packed neuroblast nuclei (Fig. 2a, c). NG2^+^ TNTs were seen to arise from the microvessel wall directed toward the facing vessel or completely bridging the gap between the vessels (Fig. 2a, c). The pericyte nature and morphological details of the detected TNTs were better demonstrated on the NG2 staining single channel (red; Fig. 2b, d). In the Additional file (Additional file 3a–f), the same cortex fields shown in Fig. 2 are shown on single, separate, staining channels (Glut-1, NG2, and GFAP), without nuclear counterstaining, in B&W, and with the addition of a bas-relief filter, to better demonstrate the exclusive origin of TNTs from pericytes (Additional file 3a, b, d, e) and their tunneling course throughout the dense, cortex parenchyma (Additional file 3c, f). Additional file 4 consists of a sequence of single optical planes (0.35 µm thick each) and the relative structural details of TNT shown in Fig. 2a. The independence of pericyte-derived TNTs from endothelial cells was confirmed by double staining with NG2 and the endothelial marker CD31/PECAM-1 (platelet–endothelial cell adhesion molecule-1) (Fig. 3a–d). On these sections, marker specificity is confirmed, together with the demonstration of ultra-long NG2^+^, pericyte-derived TNTs (Fig. 3b, c), which in some fields can be followed from one vessel-side to the opposite one (Fig. 3d). An additional movie file (Additional file 5) shows the structure of the TNT depicted in Fig. 3c in more detail: the sequence of 44 serial optical planes (0.35 µm each) allowed the TNT root to be recognized and to follow over its entire length for about 15 µm in the section depth.

### Extracellular matrix molecules and pericyte-derived TNTs

**Fig. 4:**
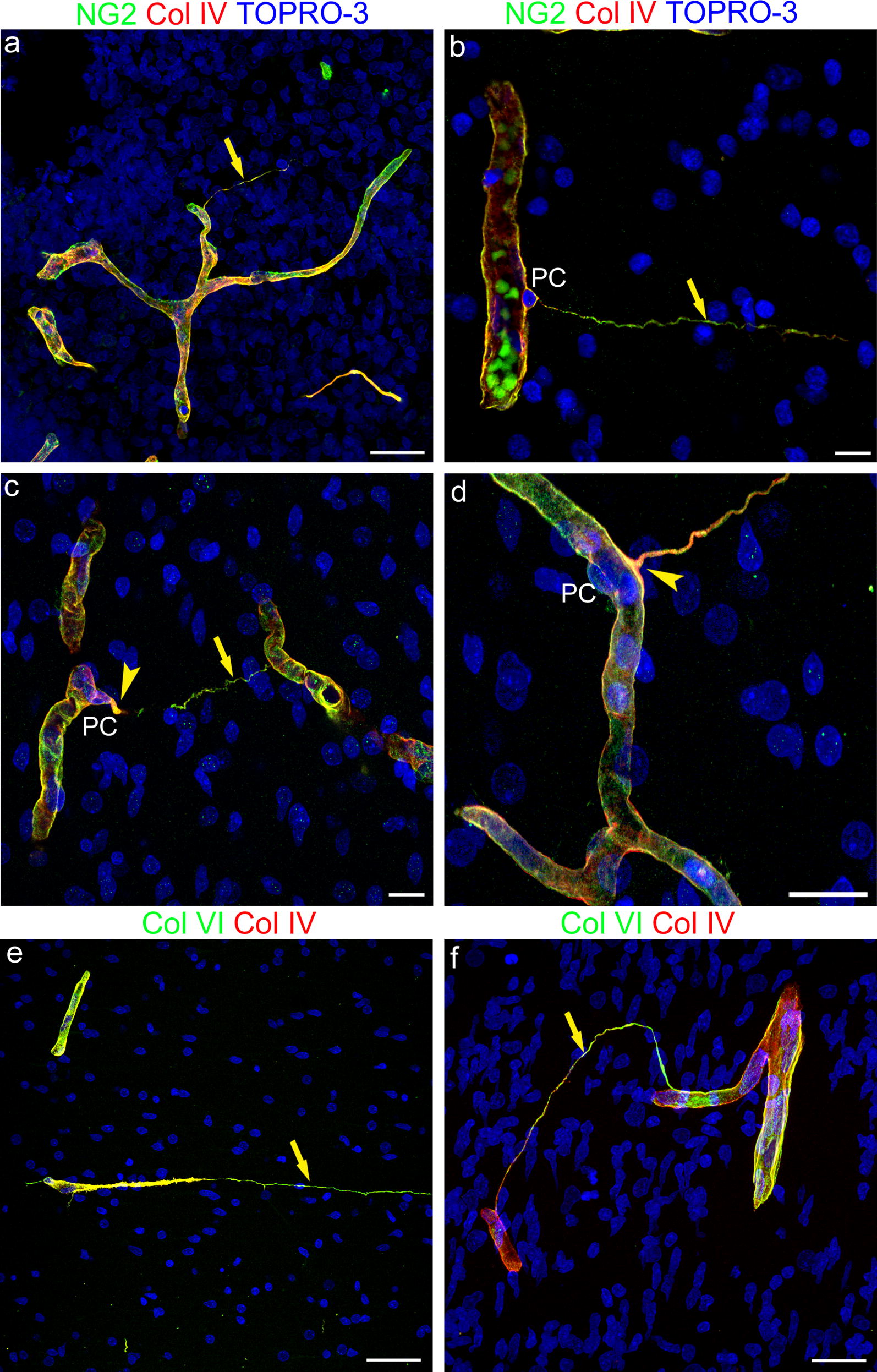
Representative confocal microscopy images of NG2/Col IV and Col VI/Col IV immunostained telencephalon sections from 18 wg (**a**, **f**) and 22 wg (**b**–**e**) fetuses. **a**–**d** NG2 and Col IV extensively colocalize on both vessel basal lamina (VBL) and TNT basal lamina (BL); NG2 staining prevails over the whole TNT extension (**a**, **b**, **c**; arrow), while Col IV appears more concentrated at the TNT root (**c**, **d**; arrowhead). **e**, **f** Col VI and Col IV extensively colocalize on both VBL and TNT BL, although Col VI seems to prevail on TNTs (arrow), closely resembling the NG2 staining. Nuclear counterstaining TOPRO-3. PC, pericyte. Scale bars **a**, **d**, **f** 25 µm; **b**, **c** 20 µm; **e** 50 µm

Further data on the structural composition were collected by analyzing TNT-associated basal lamina (BL) molecules (see the initial observations of FN^+^ TNTs; Background paragraph). Double staining with NG2 and collagen type IV (Col IV) showed colocalization of the extracellular domain of NG2 with Col IV molecules on the vessel wall and TNT surface (Fig. 4a–d). This was more evident on the TNT root (Fig. 4c, d), whereas on the far TNT extension, NG2 prevailed (Fig. 4b, c). On double staining with Col VI, that is known to bind the extracellular NG2 domain, and Col IV, the former clearly resembled the NG2 extension on the more distant TNT tracts (Fig. 4e, f).

### CD146 and NG2 identify pericyte TNTs

**Fig. 5:**
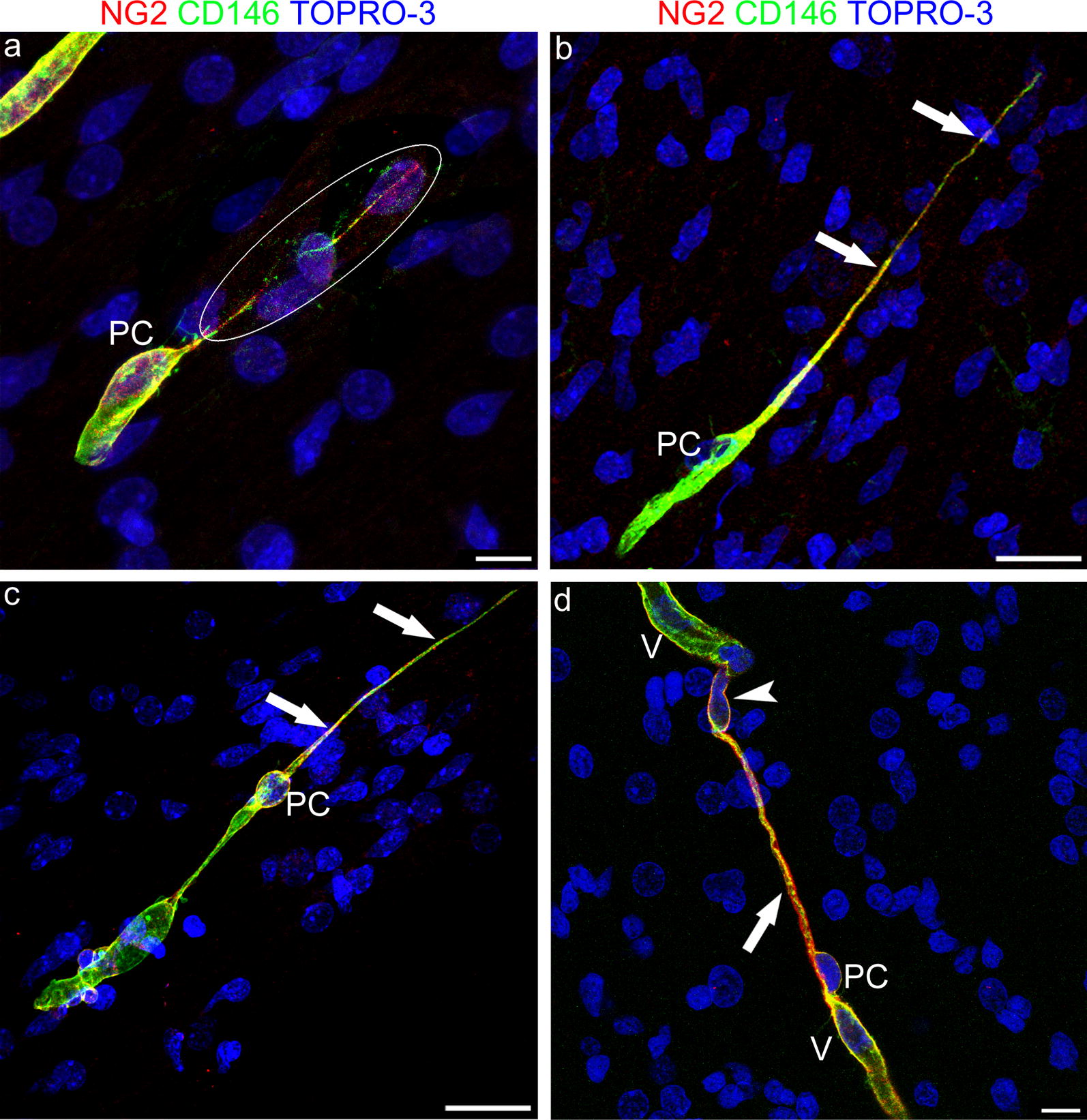
Representative confocal microscopy images of NG2/CD146 immunostained pericyte TNTs in the developing human telencephalon at 22 wg. **a** A very thin, double stained TNT, revealed by the overexposed laser confocal microscope setting (encircled area), originates from an NG2^+^/CD146^+^ pericyte (PC). **b**, **c** NG2 and CD146 extensively colocalized along the entire extension of pericytes TNTs (arrows), although on the TNT distant ends CD146 staining prevails. **d** An NG2^+^/CD146^+^ larger TNT conduit (arrow) appears to connect the tip of a growing microvessel (V), escorted by a leading pericyte (PC), with a recipient vessel (V); note a nucleus (arrowhead) that apparently fills the TNT end (see also Additional file 6). Nuclear counterstaining TOPRO-3. PC, pericyte. Scale bars **a**, **d** 10 µm; **b**, **c** 25 µm

Additional information on the molecular composition of plasma membranes involved in TNT formation was obtained with another pericyte marker, the single cell-adhesion receptor CD146 (also known as cell surface glycoprotein MUC18). On double staining with NG2, CD146 was shown to colocalize with NG2 over the entire extension of pericytes’ TNTs (Fig. 5a–d). On the finest and more distant TNT tracts, CD146 staining prevailed and NG2 was detected as a few scattered molecules on the TNT surface (Fig. 5c). Interestingly, double CD146/NG2 staining also showed larger conduits, where CD146 and NG2 were colocalized on the innermost TNT surface, while NG2, as a transmembrane molecule characterized by a large extracellular domain, also marked the outer surface (Fig. 5d). In Additional file 6, on this type of TNTs showing the sequence of single optical planes (from the projection image of Fig. 5d), we could recognize the cytoplasm inside the TNT, which seems to permit the passage of a cell nucleus (Additional file 6).

### TNTs and cerebral cortex vascularization

**Fig. 6:**
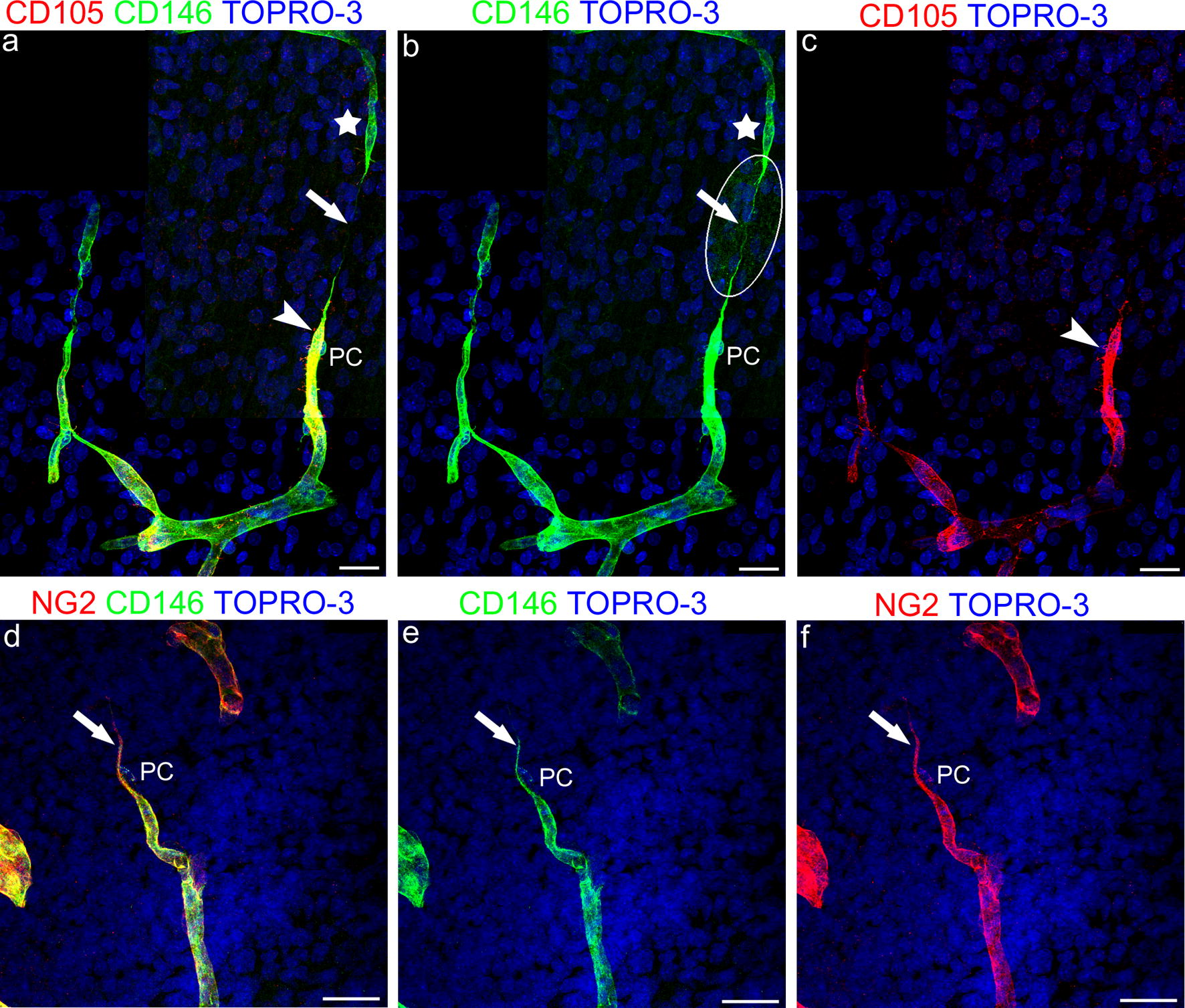
Representative confocal microscopy images of pericyte TNT involved in vessel growth and sprouting in the developing human telencephalon at 22 wg (**a**–**c**) and 18 wg (**d**–**f**). **a**–**c** CD105/CD146 and **d**–**f** NG2/CD146 double immunostainings. **a**–**c** Comparison of the merged image (**a**) with single channels (**b**, **c**) allows a ‘complete’ vessel sprout to be identified, formed by a CD146^+^ escorting pericyte (PC in **a** and **b**) and a CD105^+^ endothelial cell (arrowhead in **a**, **c**) and a CD146^+^, pericyte-like sprout (star in **a** and **b**), that does not stain positive for CD105, connected by a CD146^+^ pericyte-like TNT (arrow in **a** and **b**); the encircled area in **b** corresponds to an overexposed laser confocal microscope setting. **d**–**f** NG2 colocalized with CD146 on a leading pericyte (PC) and on its short TNT (arrow). Nuclear counterstaining TOPRO-3. Scale bars **a**–**f** 25 µm

Based on the results obtained with CD146^+^/NG2^+^ TNTs, fields of vessel growth and sprouting were searched for and analyzed utilizing CD105 (endoglin), a molecule involved in angiogenesis and specifically expressed by proliferating ECs and by sprouting endothelial tip cells (ETCs) (Additional file 7; see also [25]). On double CD105/CD146 and NG2/CD146 immunostainings (Fig. 6a–f; Additional file 7), CD146^+^ TNTs were seen to originate from the leading pericytes of two facing vessel sprouts (Fig. 6a, b), while stalk ECs and ETCs expressed high levels of CD105, although the latter did not seem to be involved in TNT formation (Fig. 6a, c), and phalanx EC were weakly stained by CD105 (Fig. 6a, c). The pericyte origin of CD146^+^ TNTs that were seen to connect vessel sprouts was confirmed by double staining, which showed CD146 to colocalize with NG2 on leading pericyte-like cells and on their short TNTs (Fig. 6d–f).

### TNTs and glioblastoma vascularization

**Fig. 7:**
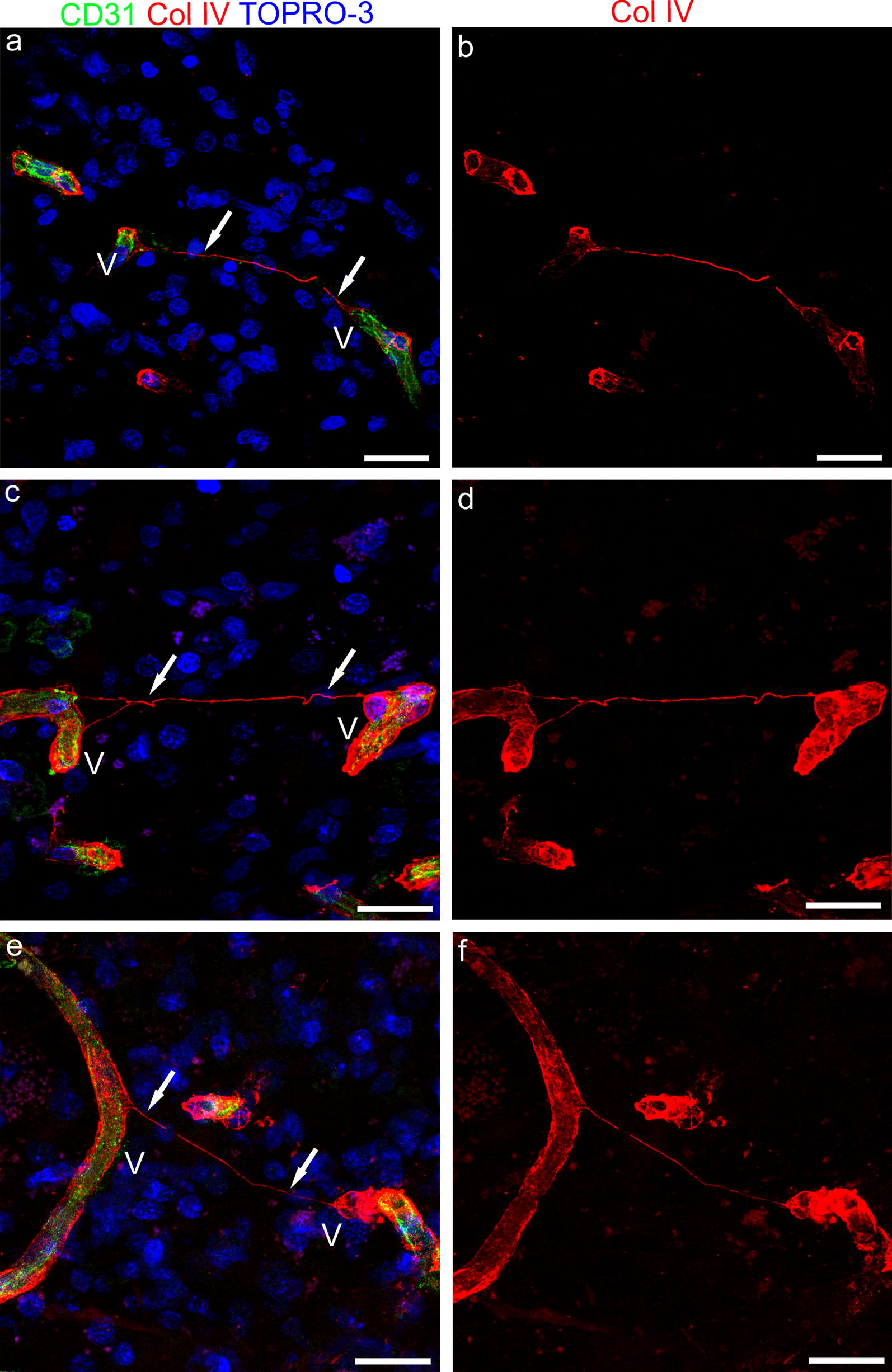
Representative confocal microscopy images of human glioblastoma sections immunostained for CD31 and Col IV. **a**, **c**, **e** Col IV^+^ TNTs (arrows) connect capillary-like tumor vessels (V); note in **c** and **e** the weak CD31 staining of tumoral endothelial cells. **b**, **d**, **f** On the single Col IV channel (red) the continuity of TNTs is better shown. Nuclear counterstaining TOPRO-3. Scale bars **a**, **b** 25 µm; **c**–**f**, 20 µm

One of the histopathological features of GBs is a prominent microvascular proliferation with an abundance of disorganized microvessels and the aggressive proliferation of ECs and pericytes. Because GBs are highly vascularized tumors, a parallel study on TNTs was conducted on human GB samples (Fig. 7a–d), to ascertain whether, as observed during normal brain vascularization, ‘vascular’ TNTs form between vessels also in pathological tumoral angiogenesis. Although ECM molecules did not allow the cell of origin of TNTs to be identified, the molecules that were used in fetal cerebral cortex samples did indeed show a very high sensitivity in revealing TNTs. For this reason and braced by the previous results, which showed a good correspondence between NG2 and Col IV, we decided to approach the study by performing double immunostaining of human GB sections with CD31 and Col IV (Fig. 7a–d). Among the different types of microvascular formations described [26, 27], tumoral microvascular sprouting has been defined as characterized by the proliferation of delicate capillary-like microvessels resembling those seen during classic angiogenesis, distributed evenly throughout the major parts of vital tumor tissue. When analyzed on CD31/Col IV stained sections, these tumoral areas showed a rich network of TNTs that extensively connected distant capillary-like tumoral vessels (Fig. 7a–f), which closely resembled the thinner forms of TNTs observed in the developing cortex.

### Pericyte TNTs in cultures

**Fig. 8:**
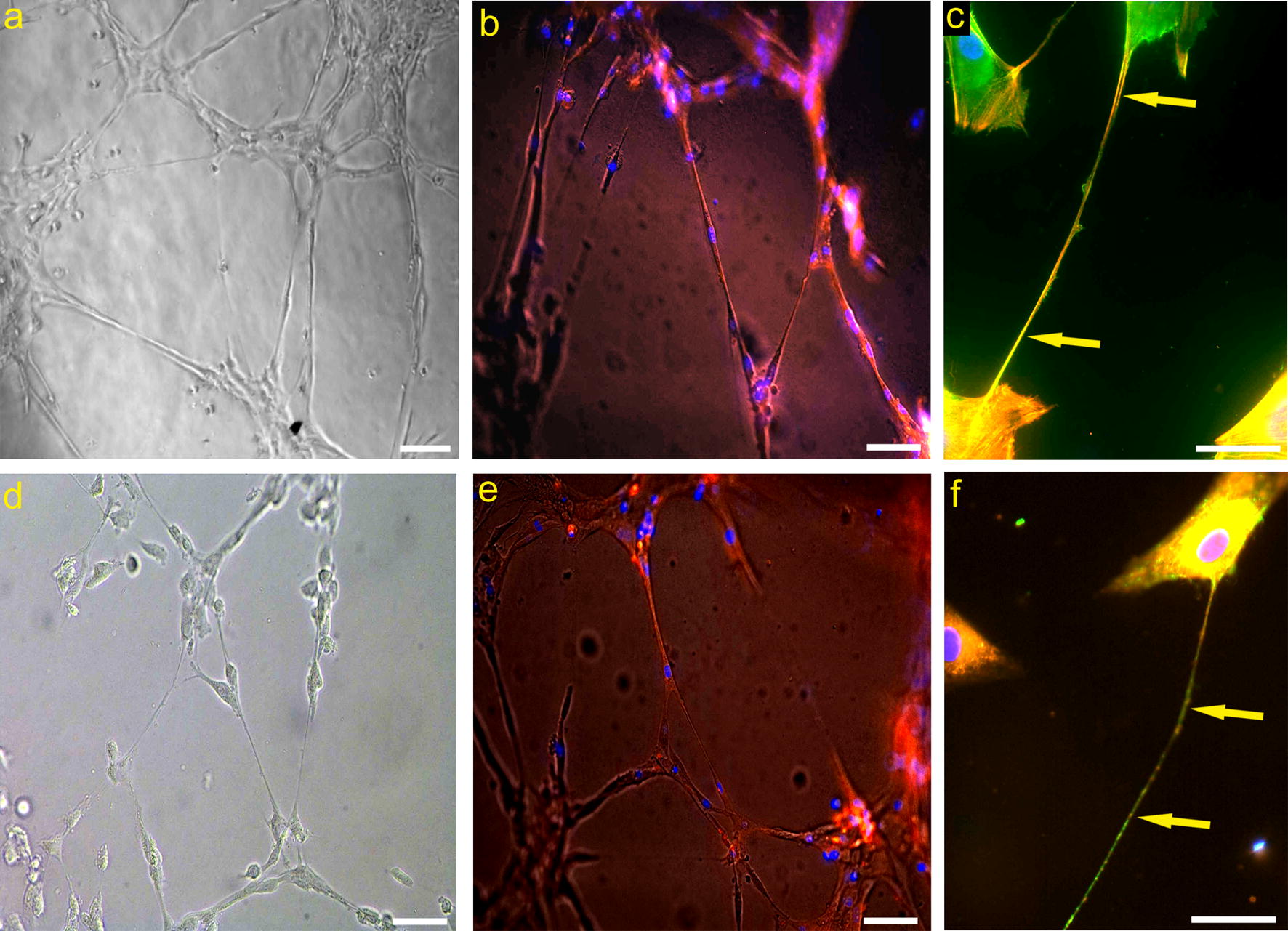
Human brain vascular pericytes (HBVP) TNTs in vitro. **a**, **d** Representative TNT networking among HBVP cultured on Matrigel layer. **b**, **e** Filaments of actin contained in HBVP-derived TNTs stained with Phalloidin TRITC-conjugated dye. **c**, **f** NG2/CSPG4 immunostaining of pericytes (green), pretreated with Phalloidin TRITC-conjugated dye (**c**, red), or with lipophilic DiI cell tracker (**f**, red), shows long NG2^+^ TNTs (arrows). Nuclear counterstaining DAPI (**b**, **c**, **e**, **f**). Scale bars **a**, **b**, **d**, **e** 50 µm; **c**, **f** 25 µm

The authenticity of the pericyte TNTs observed in the fetal brain was largely confirmed by the ability of isolated brain vascular pericytes to reproduce such structures in vitro. In fact, when pericytes were cultured in Matrigel (in conditions promoting tube formation of endothelial cells), the arrangement of pericytes and pseudo-tubular structures could be observed, demonstrating that single cells or groups of cells were frequently connected by TNT-like structures of various lengths (Fig. 8a–f). Fluorescent vital staining of the pericytes with the lipophilic dye, DiI (Fig. 8f), confirmed that the observed cell filopodial processes did indeed emanate through extensions of the plasma membrane, while Phalloidin labeling confirmed that they were sustained by actin microfilament bundles (Fig. 8b, e). Finally, immunostaining with antibodies to NG2/CSPG4 further corroborated this view, since the nanotubes were decorated by the proteoglycan (Fig. 8c, f).

### TNTs measurement in situ and in vitro

Measurements were carried out on randomly chosen, NG2 stained sections (n = 13) of fetal cerebral cortex (n = 4) for a total of 63 TNT fields. The resulting estimated thickness was 1.614 ± 0.718 µm (M ± SD), and Max and Min values were 2.9 µm and 0.34 µm, respectively. Size assessment of pericyte TNTs in in vitro assays indicated an average thickness of 1.88 ± 0.25 μm.

## Discussion

It has been demonstrated that pericytes contribute to the barrier phenotype of brain ECs and that they are involved in the formation and regulation of the BBB [28, 29]. It is also well known that vascularization of the central nervous system (CNS) occurs via an angiogenic mechanism of vessel sprouting from the pre-existing vessels of the perineuronal vascular plexus and from the perforating, intraparenchymal, radial vessels [30, 31]. It has been underlined in several studies that pericytes are recruited through classic PDGF/PDGFR-β-mediated signaling, after the active phase of vessel sprouting, mainly directed by stalk and tip endothelial cells, having the effect of stabilizing the growing vessel, favoring EC BBB differentiation [32]. On the other hand, it has also been observed that in pathological conditions, as well as in normal developing CNS, NG2^+^/PDGFR-β^+^ pericytes closely cover CD31^+^ ECs, spanning the gap between neighboring endothelial cell sprouts, being tightly associated with CD31^+^ ETCs [33, 34]. In their study [33], the Authors stressed the need for ‘more detailed investigation in future work’ on the detected ‘putative pericyte bridges’. Immature pericytes have been described in association with the tips of capillary sprouts in the mesenteric microvascular bed [35] and also seen at and in front of the advancing tips of endothelial sprouts, as well as bridging the gap between the leading edges of opposing endothelial sprouts which were apparently preparing to merge [36]. We have previously demonstrated that during fetal brain vascularization, NG2^+^/PDGFR-β^+^ angiogenic/activated pericytes seem to be involved in the early phases of angiogenesis, appearing in extensive association with the telencephalon vasculature, and in intimate contact with vascular sprouts [37]. This suggests that during CNS angiogenesis, the sprouting phase of nascent vessels is controlled by an intimate interplay between escorting pericytes and ETCs and that guiding (pioneering) pericytes may initiate the angiogenic process by forming strands connecting to existing capillaries [37, 38]. More recently, pericytes have been suggested to promote endothelial sprouting by producing hepatocyte growth factor (HGF) [39], and the hypothesis has been advanced that like stalk and tip endothelial cells, so also pericytes can recognize different subpopulations that accomplish different, specific roles, including making an active contribution to endothelial sprouting [40]. Certainly pericyte subpopulations exist, also stemming from different cell precursors, and the origin of forebrain pericytes from cephalic neural crest cells [41] may explain the mesenchymal potential and phenotypic and functional singularity of brain pericytes, including their participation in the earliest stages of neovascularization [42, 43]. The searching, guiding, and communicating activities played by pericyte-derived TNTs while aiding the outgrowth of endothelial cells may be included in this scenario.

### TNTs and Basal lamina molecule relationships

Intriguingly, extending nanotubes appear fully enwrapped by a complex ECM, composed by fibronectin, Col IV and Col VI. Pericytes have been identified as important players in regulating basement membrane organization, in particular the deposition, during development, of fibronectin, laminin and collagen IV, which may be key molecules in regulating pericyte-ECs connections [43]. Pericyte TNTs carry the transmembrane proteoglycan NG2/CSPG4, and the link between the large extracellular domain of this molecule and Col VI [44] may contribute to TNTs’ basal lamina (BL) organization. The BL molecules associated to TNTs may provide mechanical support to the extending cell nanostructures, and also mediate signaling and mechanical strains [45] between cells over long distances.

### Pericyte TNTs at point of vessel sprouting and vessel collateralization

It could be suggested that the reason for a precocious involvement of pericyte TNTs during angiogenesis is to allow an indispensable cell-to-cell recognition and communication during both physiological (developing brain) and pathological (glioblastoma) neovascularization. During the early phases of vessel growth, pericyte TNTs may send molecular messages and/or organelles to the receiving cell (pericyte, EC, or other?) in the targeted vessel (e.g. between two distant vessels) or originating from an ETC-escorting pericyte, may guide sprout-to-vessel and sprout-to-sprout recognition and crosstalk. In addition, TNTs were also seen bridging the gap between CD146^+^/CD105^−^ and CD146^+^/CD105^+^ vessel sprouts. This observation closely resembles the long ‘endothelial’ filopodia described by Thomas Bär (see Additional file 1). The nature of the sprout-connecting long filopodia can be discussed in the light of the recent data gained on CD146 dynamic expression in ECs and pericytes during brain development. At this stage CD146 has been demonstrated to be expressed by ‘naked’, immature ECs, while in microvessels covered with pericytes CD146 appeared exclusively expressed by pericytes, which are induced to express high levels of CD146 in parallel with a TGF-β pericyte-derived downregulation of CD146 in ECs [46]. Interestingly, Cd146-KO mice and Cd146^PC^-KO, compared with Cd146^EC−^KO, showed a reduction in pericyte coverage and an abnormal BBB development (abnormal tight junctions/claudin-5 expression). In fact, CD146, which is constitutively expressed by brain pericytes, appeared to be involved in pericyte recruitment, working as a coreceptor in the PDGF-B/PDGFR-β signaling [46]. CD105/endoglin is a homodimeric transmembrane glycoprotein that is strongly expressed on endothelial precursor cells [47] and angiogenically activated endothelial cells, and is involved in vascular development and remodeling [48], showing a 50% reduced ratio with PECAM in normal vessels of newborn brain [49]. In fact, at the protein level, during fetal brain angiogenesis CD105 appears strongly expressed by ETCs, thus making the typical ETC’s filopodial protrusions recognizable (Additional file 7; see also [25]). Although it is claimed that no surface markers exclusively expressed by pericytes exist [50], pericytes can be identified by coexpressing canonical pericyte markers, CD146 and NG2 [51]. Together with the absence of CD105, these data strongly suggest the existence in brain tissue, in vivo, of sprouting cells with a pericyte-like phenotype, and of couples of hetero-sprouts, involved in vessel branching and possibly connected by pericyte TNT-like cell protrusions. This is consistent with previous observations that assigned a leading role to pericytes in vessel sprouting during human cerebral cortex development, and demonstrated pericytes initiating sprouting, and forming long strands between existing capillaries [38].

### TNTs vs microtubes (MT): simple cousins or connected structures?

Double NG2/CD146 staining also showed a larger subset of pericyte TNTs, that can be regarded as the big cousins of the classical tiny structures. As suggested for tumor microtubes (TMs), the latter likewise promote tumor aggressiveness and therapeutic resistance [21, 52]. In vivo microscopy methods made it possible to discover highly dynamic, large and long (cross-sectional area 1.57 ± 0.33 µm^2^, and more than 500 µm in length) glioblastoma cell TNT-like protrusions, TMs, scanning the surrounding microenvironment, carrying mitochondria and microvesicles and, after mitosis, traveling daughter cell nuclei [52, 53]. Although only based on ‘static’ confocal microscopy images rather than live imaging, the hypothesis may be advanced that the large, ultra-long TNTs observed during normal fetal neuroangiogenesis, and seen to host cell nuclei, are possibly channeling the nucleus of daughter pericytes (see Additional file 6).

### Pericyte TNTs in pericyte-to-pericyte and pericyte-to-endothelial cell communication

We have previously demonstrated that in human GB, NG2 and Col IV are extensively colocalized on pericyte layer/s of both capillary-like microvessels and glomeruloid structures [24]. In the present study, Col IV^+^/CD31^−^ TNTs have been demonstrated, connecting tumoral vessels marked by endothelial CD31, suggesting that as observed in normal brain vascularization, ‘vascular’ TNT nanostructures that span the gap between tumoral vessels, can likewise be involved in pathological tumoral angiogenesis. Interestingly, membrane protrusions identified as TNTs with a mural cell (smooth muscle cells and pericytes) origin have been shown to be able to transfer microRNAs to ECs and modulate their angiogenesis and vessel stabilization properties [54]. Origin and ‘recipient’ cells of the observed GB TNTs need to be further investigated, although if they are considered in parallel with TNTs observed during normal angiogenesis, and in the light of in vitro studies a specific role can be postulated also in tumor angiogenesis. Interestingly, recent studies conducted by confocal live cell imaging of myeloid-derived cells in corneal explants from Cx(3)cr1(GFP) and CD11c(eYFP) transgenic mice have revealed that, unlike in *in* vitro studies, where nanotubes originate from cell–cell contacts, in in vivo tissue, these structures form de novo as a response to specific ‘stress’ factors at a rate of 15.5 μm/min [55]. These results support our thesis that TNTs may also be elicited by growth factors involved in angiogenesis events. Equally relevant findings, for the scope of our study, emerge from the analysis of TNT-dependent electrical coupling between developing embryonic cells, and the potential implication of this TNT role in cell-to-cell signaling in developmental processes [56]. It must not be forgotten that direct pericyte–endothelial contact is established via junctional complexes located at peg–socket contacts, which also contain gap junctions [see for a review on ‘Endothelial/Pericyte Interactions’ Armulik et al. [43]. Therefore, it cannot be excluded that blind-ended TNTs provided with gap junction components may mediate pericyte-endothelial communication between distant, growing vessels.

## Conclusions

Overall, the present data provide further evidence and different examples of TNTs and/or TNT-like microstructures in human brain development and in human GB, adding another tile to the documented existence of TNTs in tissues in vivo (see Ariazi et al. for a recent Review [57]). Moreover, our findings reveal a possible novel role for brain pericytes that, by giving origin to TNTs, may be involved in the earliest phases, in searching, recognition, connection, and communication, during normal and pathological angiogenesis, thus stressing the leading role of pericytes demonstrated in previous studies [37, 38]. Metastatic invasiveness has recently been associated to TNT horizontal transfer of miRNA between metastatic cancer cells and endothelial cells [58]. Pericyte TNTs in glioblastoma may open out the vista of a possible TNT-based communication between vessel cell components (pericytes, endothelial cells), which may contribute to the suggested MT-dependent chemotherapy resistance [12, 18, 19, 52].

## Additional files

**Additional file 1.** Formation of TNTs during the process of vessel sprouting. a Part of the original diagram from Bär [2] that summarizes the formation of net-capillaries in the cerebral cortex; the step denoted ‘fusion’ is described as *“A fusion of capillary sprouts with preexisting capillaries or with another sprout may be initiated by contact of small endothelial tentacles.”* (Springer Nature License to Daniela Virgintino, Bari University School of Medicine). b Similar facing sprouts connected by a TNT are revealed by confocal microscopy and CD146/CD105 immunostaining in the fetal cerebral cortex (see also Fig. 6 and the Result paragraph). Scale bar b, 25 µm.

**Additional file 2.** Additional examples of straight and spiraled TNTs immunostained with fibronectin (FN). a Two parallel, tiny and ultra-long FN^+^ TNTs (*arrows*), whose continuity is revealed in the FN single channel (b). c, d A typical bridging TNT (*arrows*) e, f A TNT characterized by an irregular course (*arrow*), clearly originates from a pericyte (PC). Nuclear counterstaining propidium iodide (PI). Scale bars a, b 50 µm; c–f 25 µm.

**Additional file 3.** A single channel, grayscale, bas-relief image of Fig. 2a, c. The digital filters applied to pictures a and b in Fig. 2, highlight the endothelial profile (a, d), the pericyte origin of TNTs (b, e), and their excavated course (tunneling) through the dense, fetal cerebral cortex parenchyma (c, f; *arrows*). Scale bars a, b 25 µm; c, d 20 µm.

**Additional file 4.** Single optical planes show TNT details and relationship. Sequence of single optical planes from the z-stack of Fig. 2a.

**Additional file 5.** Following the TNT deep into section thickness. TNT depicted in Fig. 3c (projection image) results from the sequence of 44 optical planes through the entire section thickness (about 15 µm).

**Additional file 6.** Does the large TNTs convey cell nuclei? Sequence of single optical planes from the z-stack of Fig. 5d, shows the TNT plasma membrane and a cell nucleus inside the TNT.

**Additional file 7.** CD105 is a marker of angiogenically activated endothelial tip cells in the fetal cerebral cortex. a–c Examples of CD105^+^ activated. d–f Examples of CD105^+^ endothelial cells, covered by CD146^+^ pericytes (PC). Nuclear counterstaining TOPRO-3. Scale bar a, b, d 20 µm; c, e, f 10 µm.

## Abbreviations

BB: blocking buffer
BBB: blood–brain barrier
BL: basal lamina
CNS: central nervous system
Col IV: collagen type IV
Col VI: collagen type VI
DAPI: 4′,6-diamidino-2-phenylindole
ECM: extracellular matrix
EC: endothelial cell
ETC: endothelial tip cell
FBS: fetal bovine serum
FCS: fetal calf serum
FN: fibronectin
GB: glioblastoma
GFAP: glial fibrillary acidic protein
Glut-1: glucose transporter isoform 1
HBVP: human brain vascular pericytes
mAb: monoclonal antibodies
NG2/CSPG4: neuron-glial antigen 2/chondroitin sulphate proteoglycan 4
pAb: polyclonal antibodies
PBS: phosphate-buffered saline
PCM: pericyte culture medium
PDGF-B: platelet-derived growth factor subunit B
PDGFR-β: β-type platelet-derived growth factor receptor
PECAM-1: platelet endothelial cell adhesion molecule 1
PFA: paraformaldehyde
PI: propidium iodide
RNase: ribonuclease
TGF-β: transforming growth factor β
TNT: tunneling nanotube
TRITC: tetramethylrhodamine-isothiocyanate
VBL: vessel basal lamina
wg: weeks of gestation
