# Supplementary figures and images for: Tunneling nanotubes evoke pericyte/endothelial communication during normal and tumoral angiogenesis
Source: Fluids Barriers CNS. 2018 Oct 5;15:28. doi: 10.1186/s12987-018-0114-5 (PMC6173884; doi:10.1186/s12987-018-0114-5)

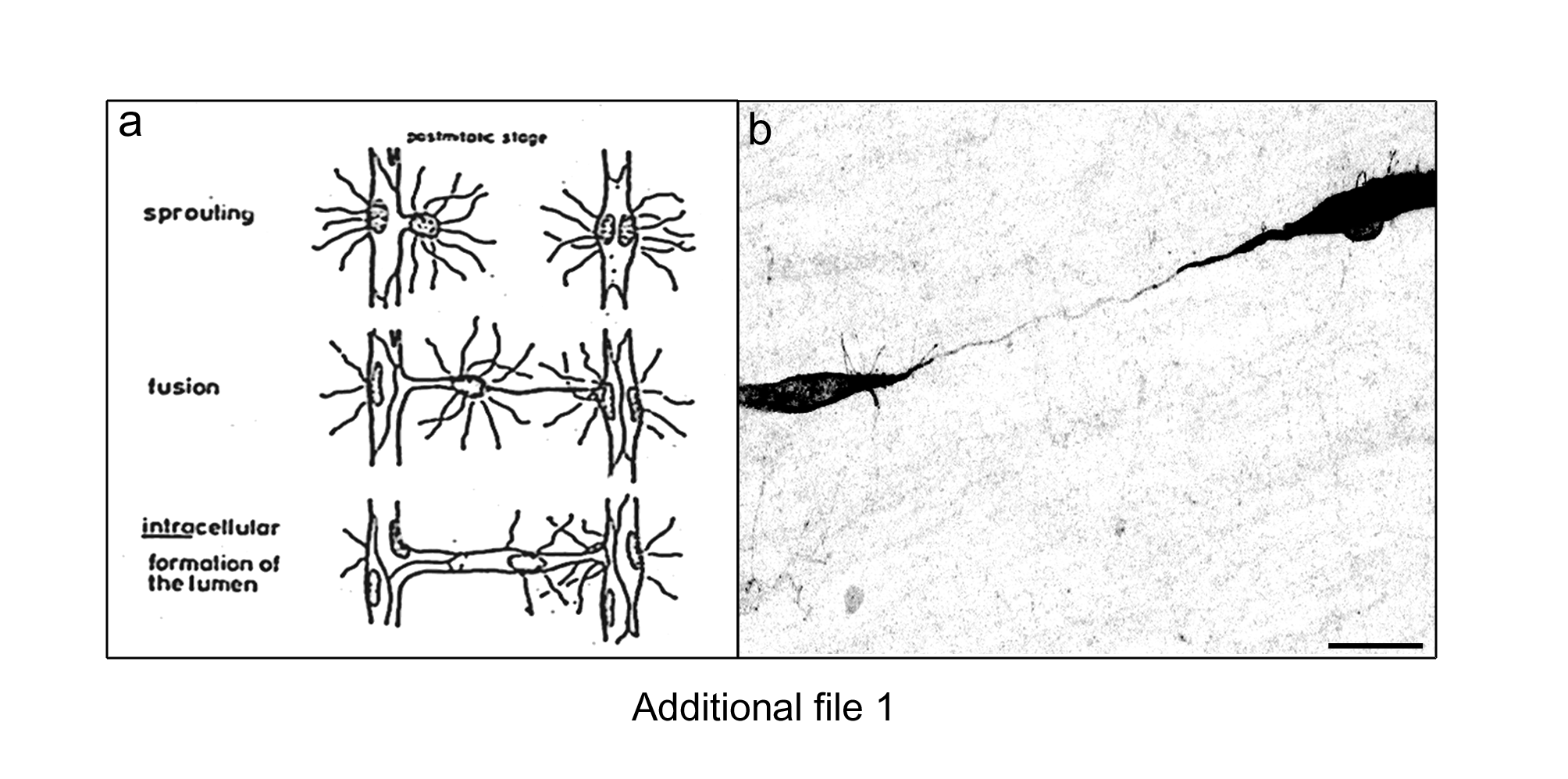

Supplement: Supplementary file 1 — Additional file 1. Formation of TNTs during the process of vessel sprouting. a Part of the original diagram from Bär [2] that summarizes the formation of net-capillaries in the cerebral cortex; the step denoted ‘fusion’ is described as “A fusion of capillary sprouts with preexisting capillaries or with another sprout may be initiated by contact of small endothelial tentacles.” (Springer Nature License to Daniela Virgintino, Bari University School of Medicine). b Similar facing sprouts connected by a TNT are revealed by confocal microscopy and CD146/CD105 immunostaining in the fetal cerebral cortex (see also Fig. 6 and the Result paragraph). Scale bar b, 25 µm. [file 12987_2018_114_MOESM1_ESM.tif]

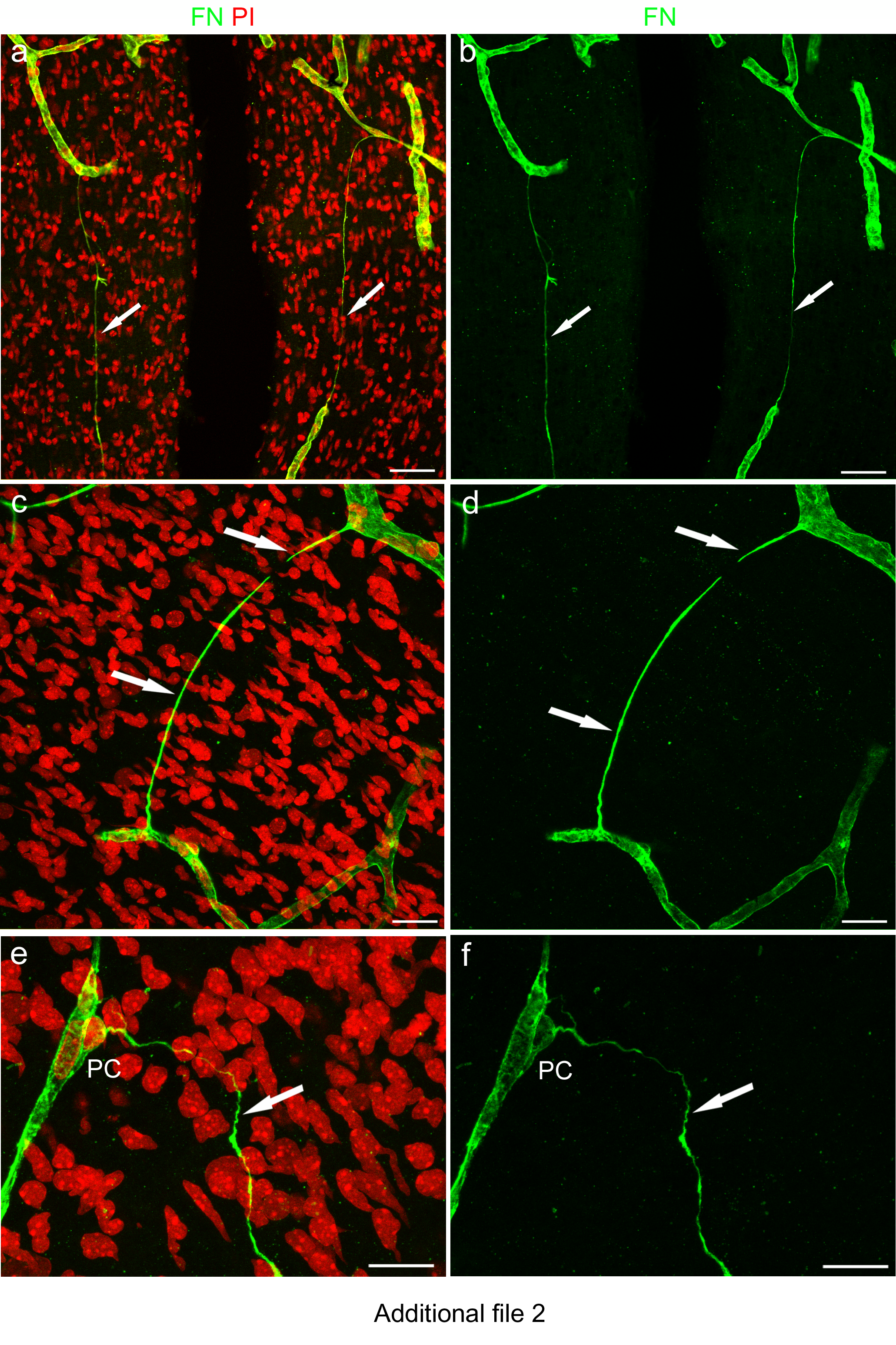

Supplement: Supplementary file 2 — Additional file 2. Additional examples of straight and spiraled TNTs immunostained with fibronectin (FN). a Two parallel, tiny and ultra-long FN+ TNTs (arrows), whose continuity is revealed in the FN single channel (b). c, d A typical bridging TNT (arrows) e, f A TNT characterized by an irregular course (arrow), clearly originates from a pericyte (PC). Nuclear counterstaining propidium iodide (PI). Scale bars a, b 50 µm; c–f 25 µm. [file 12987_2018_114_MOESM2_ESM.tif]

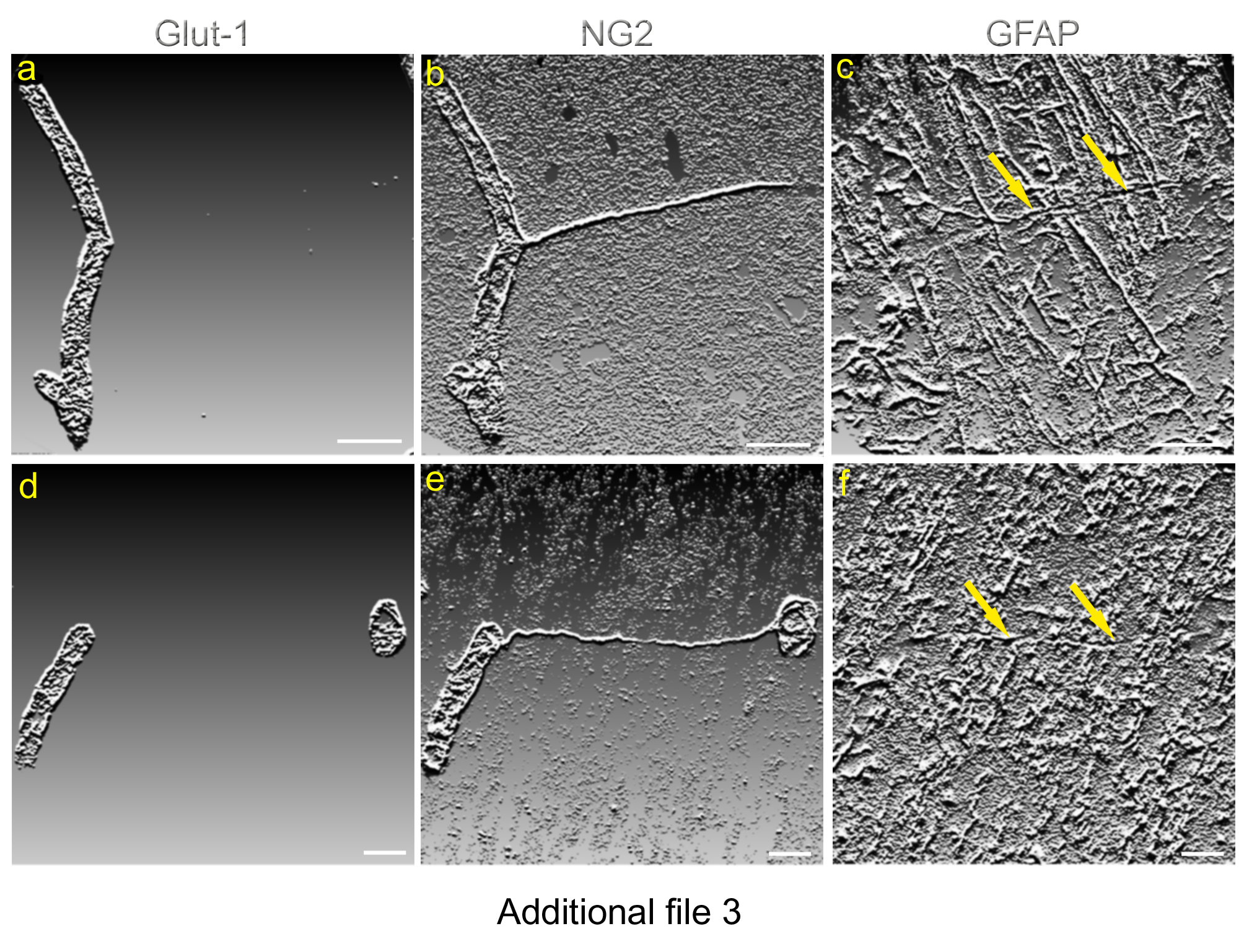

Supplement: Supplementary file 3 — Additional file 3. A single channel, grayscale, bas-relief image of Fig. 2a, c. The digital filters applied to pictures a and b in Fig. 2, highlight the endothelial profile (a, d), the pericyte origin of TNTs (b, e), and their excavated course (tunneling) through the dense, fetal cerebral cortex parenchyma (c, f; arrows). Scale bars a, b 25 µm; c, d 20 µm. [file 12987_2018_114_MOESM3_ESM.tif]

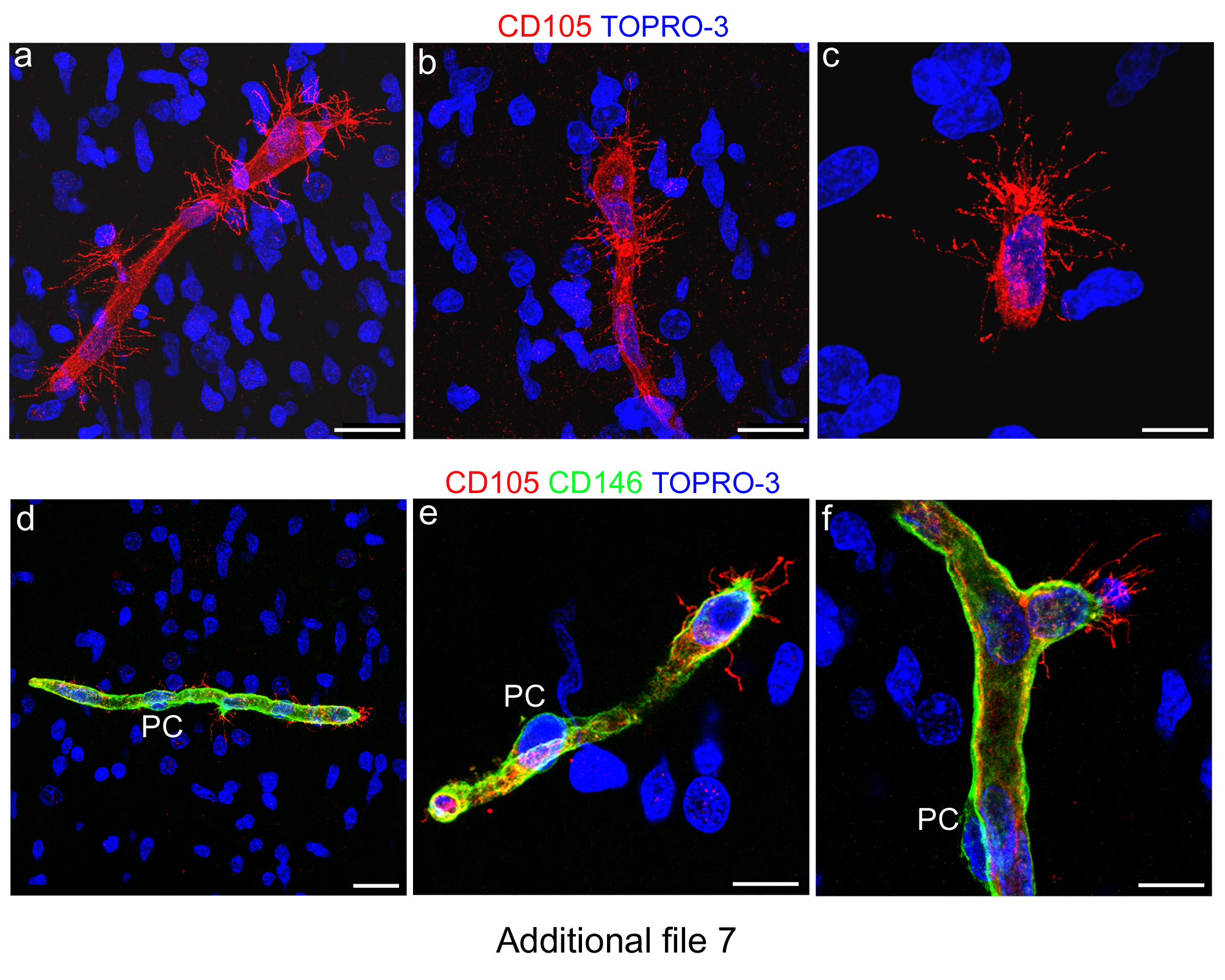

Supplement: Supplementary file 7 — Additional file 7. CD105 is a marker of angiogenically activated endothelial tip cells in the fetal cerebral cortex. a–c Examples of CD105+ activated. d–f Examples of CD105+ endothelial cells, covered by CD146+ pericytes (PC). Nuclear counterstaining TOPRO-3. Scale bar a, b, d 20 µm; c, e, f 10 µm. [file 12987_2018_114_MOESM7_ESM.tif]
